# Characterizing Human Disparity Tuning Properties Using Population Receptive Field Mapping

**DOI:** 10.1523/JNEUROSCI.0795-24.2025

**Published:** 2025-02-07

**Authors:** Ivan Alvarez, Alessandro Mancari, I. Betina Ip, Andrew J. Parker, Holly Bridge

**Affiliations:** ^1^Oxford Centre for Functional MRI of the Brain (FMRIB), Wellcome Centre for Integrative Neuroimaging, University of Oxford, Oxford OX3 0LN, United Kingdom; ^2^Nuffield Department of Clinical Neurosciences, University of Oxford, Oxford OX3 0DU, United Kingdom; ^3^Department of Psychiatry, University of Oxford, Oxford OX3 7JX, United Kingdom; ^4^Department of Biological Sciences, Scuola Normale Superiore, Pisa 56126, Italy; ^5^Department of Physiology, Anatomy and Genetics, University of Oxford, Oxford OX1 3PT, United Kingdom; ^6^Laboratory of Sensory Physiology, Institute of Biology, Otto-von-Guericke University, Magdeburg 39106, Germany

**Keywords:** binocular disparity, disparity tuning, MRI, population receptive field, psychophysics

## Abstract

Our visual percept of small differences in depth is largely informed by binocular stereopsis, the ability to decode depth from the horizontal offset between the retinal images in each eye. While multiple cortical areas are associated with stereoscopic processing, it is unclear how tuning to specific binocular disparities is organized across the human visual cortex. We used 3 T functional magnetic resonance imaging to generate population receptive fields (pRFs) in response to modulation of binocular disparity to characterize the neural tuning to disparity. We also used psychophysics to measure stereoacuity thresholds compared with backgrounds at different depths (pedestal disparity). Ten human participants (seven females) observed correlated or anticorrelated random-dot stereograms with disparity ranging from −0.3 to 0.3°, and responses were modeled as one-dimensional tuning curves along the depth dimension. First, we demonstrate that lateral and dorsal visual areas show the greatest proportion of vertices selective for binocular disparity. Second, with binocularly correlated stimuli, we show a polynomial relationship between preferred disparity and tuning curve width, with sharply tuned disparity responses at near-zero disparities, and broader disparity tuning profiles at near or far disparities. This relationship held across visual areas and was not present for anticorrelated stimuli. Finally, the individual thresholds for psychophysical stereoacuity at the three different pedestal disparities were broadly related to pRF tuning width in area V1, suggesting a possible limit for fine stereopsis at the earliest level of cortical processing. Together, these findings point to heterogeneity of disparity processing across human visual areas, comparable with nonhuman primates.

## Significance Statement

Binocular disparity arises from the horizontal separation of the two eyes and provides information for determining depth and 3D structure. We used functional magnetic resonance imaging and population receptive field mapping to measure tuning of multiple visual areas to binocular disparity in the human visual cortex. We additionally measured psychophysical thresholds for detecting binocular disparity and correlated these with the neural measures. The width of the disparity tuning was related to the preferred disparity across all visual areas. Disparity tuning widths in V1 were also related to psychophysical thresholds. These findings in the human are broadly comparable with nonhuman primates.

## Introduction

Binocular stereopsis, the ability to decode depth structure from horizontally offset retinal images, is dependent on the selectivity of visual cortex neurons to binocular disparity. Disparity selectivity first arises in the primary visual cortex (V1) where the input from the two eyes is combined (Barlow et al., 1967; Nikara et al., 1968; Pettigrew et al., 1968). These binocular neurons can exhibit “selectivity” to disparity such that each neuron has a limited range of disparities which will elicit changes in its firing rate, with a preferred disparity eliciting a maximal response (Poggio et al., 1988). While disparity tuning appears for the first time in V1, disparity tuned neurons are found throughout the visual cortex (for a review, see Parker, 2007).

Electrophysiological studies have demonstrated that neurons in nonhuman primate V1 encode a wide range of preferred binocular disparities and can show both narrow and broad tuning to disparity values (Cumming and DeAngelis, 2001; Prince et al., 2002a,b). While there does not appear to be a tight topographic organization for disparity in V1, such as that seen for ocular dominance or receptive field position, the width of the tuning curve increases with the eccentricity of the receptive field, both in V1 and in extrastriate visual areas (Prince et al., 2002a; DeAngelis and Uka, 2003; Parker, 2007; Anzai et al., 2011).

Although V1 neurons exhibit narrow disparity tuning, necessary for performing perceptual discrimination based on disparity information (Prince et al., 2002b), their responses do not predict stereoscopic depth perception (Parker, 2007). Specifically, they do not encode relative disparity between neighboring visual features, which are readily apparent to an observer (Cumming and Parker, 1999), and they reliably respond to anticorrelated patterns that encode disparity but do not produce a stereoscopic percept for the observer (Cumming and Parker, 1997).

In the human visual system, responsiveness to disparity has been demonstrated using fMRI in a wide range of human visual areas, with heterogeneity in the level of sensitivity displayed across areas (Backus et al., 2001; Neri et al., 2004; Bridge and Parker, 2007; Minini et al., 2010; Ban et al., 2012; Goncalves et al., 2015). Previous studies have aimed to relate disparity sensitivity to perceptually relevant processes. For example, responses to stereoscopic stimulation in dorsal visual areas predict the subjective percept of three-dimensional shape (Welchman et al., 2005; Chandrasekaran et al., 2007), slant (Murphy et al., 2013; Ban and Welchman, 2015), plane depth (Goncalves et al., 2015), and motion from depth (Rokers et al., 2009). In addition, several studies have mapped out disparity tuning spatially across the cortical surface in V2, V3 (Nasr et al., 2016; Nasr and Tootell, 2018), V3A (Goncalves et al., 2015), and V4 (Fang et al., 2019).

Much of the neurophysiological work involves measuring disparity tuning curves to determine not only the preferred disparity but also the width of tuning. Tuning width reflects the sensitivity to changes in disparity, which have been shown to link to stereoscopic depth discrimination (Prince et al., 2000). The population receptive field (pRF) in humans has been used across various domains but classically allows the computation of a spatial receptive field for each voxel in an fMRI image of the visual cortex (Dumoulin and Wandell, 2008; Wandell and Winawer, 2015; Benson et al., 2018). A similar procedure can be applied to the depth dimension. In this case, a stimulus is moved forward and backward in depth to allow modeling of the preferred depth for each fMRI voxel. A one-dimensional Gaussian fit to such data will provide both a preferred disparity and the width of the sensitivity to depth. This approach therefore allows, for the first time, investigation of disparity tuning across retinotopically mapped visual areas.

The current study used pRF mapping in depth to determine the distribution and width of disparity tuning in the human visual cortex. These neural measures, extracted from the pRF model, were then compared with stereoacuity measured psychophysically in the same participants. Tuning width was consistently larger for voxels with near or far (rather than zero) disparity tuning across all visual areas. In V1, there was a correlation between the pRF size and psychophysical stereoacuity measured at broadly comparable disparities. Thus, we provide the first evidence in the human visual system for a relationship between disparity tuning preference, tuning width and psychophysics building on earlier studies in the nonhuman primate (Prince et al., 2000).

## Materials and Methods

### Participants

Ten healthy participants with normal or corrected-to-normal vision took part in the study (age range 19–45 years, mean age 31.40 years, seven females). Participants were screened for normal visual acuity (Snellen chart at 6 m, <20/20 corrected) and stereoscopic vision [TNO test, correct detection at <60 arcsec; Frisby stereotest (http://frisbystereotest.co.uk/) correct detection at <40 arcsec]. Each participant took part in four sessions, two MRI sessions for main data acquisition, one MRI session for retinotopic mapping, and one for psychophysics outside of the scanner. Three out of the ten participants had retinotopic data available from a prior study and therefore only completed two MRI sessions. The study received ethical approval from the University of Oxford Central University Research Ethics Committee (R53110/RE002) and was conducted in accordance with the Declaration of Helsinki.

### fMRI estimation of disparity tuning curves

#### fMRI stimulus presentation

Visual stimuli were generated in MATLAB (v8.0, MathWorks) using Psychtoolbox (v3.0, http://psychtoolbox.org; Brainard, 1997; Pelli, 1997) and delivered via an MRI-compatible LCD display (BOLDscreen 32, Cambridge Research Systems; minimum luminance, 1.26 cd/m^2^; maximum luminance, 328 cd/m^2^). The participant viewed the display through a custom mirror stereoscope consisting of two pairs of mirror surfaces increasing the interocular distance and an additional mirror surface reflecting the image 90° for viewing by the supine participant (Ip et al., 2022). Paired images for the left and right eye were presented side-by-side in a single LCD display and aligned with the stereoscope to allow binocular fusion. A medial divider spanning the length of the scanner bore was positioned to exclude the left image reaching the right eye and vice versa. Each monocular display spanned 10.23 × 12.88° of the visual angle. All stimuli were presented binocularly, and participants confirmed that they saw stimuli in depth prior to proceeding to the main experiment.

**Figure 1.**
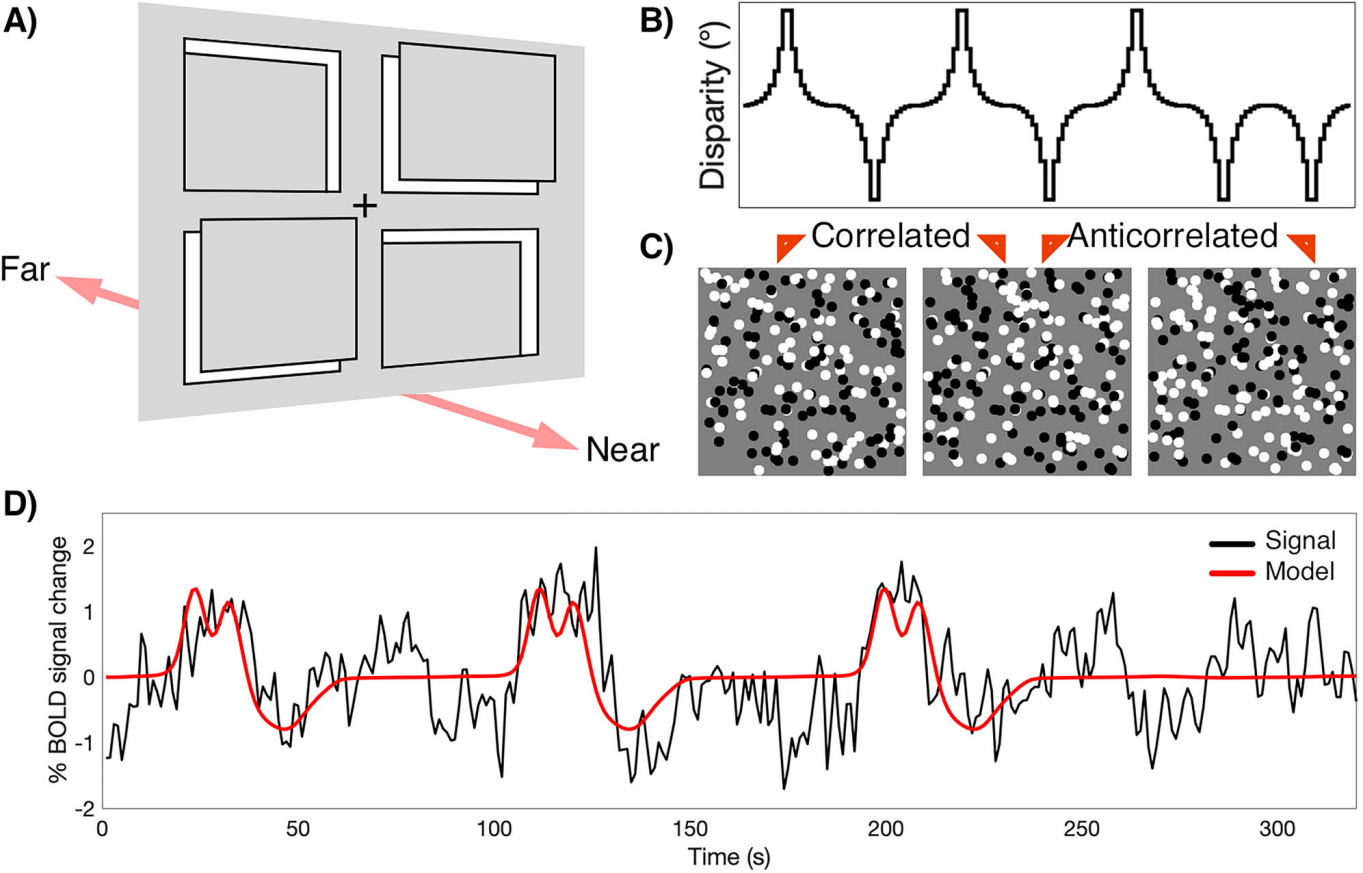
Experimental design for in-scanner binocular disparity stimulation. ***A***, The stimulus consisted of a zero-disparity background and four rectangular apertures defined by modulated disparity, positioned in front of or behind the background plane. On any given frame, two apertures displayed identical magnitude of positive disparity (near) and two apertures displayed negative disparity (far). Matched disparity patches were presented at any position, not necessarily the diagonals shown in the figure. Throughout the experiment, participants fixated on a fixation cross plotted centrally on the zero-disparity plane and performed a detection task on brief random changes in contrast within the apertures. ***B***, For any given aperture, disparity was ramped pseudorandomly, creating independent regressors. A total of 20 discrete disparity steps were shown in each cycle, spanning −0.3° to +0.3°. Each stimulus run was constructed of a series of these, each corresponding to a full disparity oscillation with a pseudorandom sign to ensure that the disparity across the four quadrants summed to zero. ***C***, The RDS stimuli were either correlated, where dot contrast was matched between the two eyes or anticorrelated in which dot contrast was opposite in the two eyes. ***D***, BOLD signals obtained under disparity stimulation were modeled with the matching disparity regressor using a one-dimensional Gaussian pRF model. The example shows the BOLD signal time course and pRF model fit for a single vertex of an example participant from area V1.

Stimuli consisted of dynamic random-dot stereograms (RDS); each frame of the stimulus contained a fixation point (0.2° radius) and 5,000 binocularly correlated black and white dots (dot radius, 0.05°; dot refresh rate, 30 Hz; 100% contrast). The dots were divided into a zero-disparity background and four disparity-defined apertures, arranged in quadrants, each spanning 3.32 × 4.64° within the RDS (Fig. 1). Each aperture was modulated separately, with binocular disparity varying in 20 log-scaled steps spanning −0.3° to +0.3° disparity from the fixation plane. The range was selected to ensure that all stimuli were easily within fusional range and to provide the greatest sampling at small disparity values. To reduce the drive to converge on the depth plane of the stimulus, on a given frame where binocular disparity was present, any two quadrants displayed a near disparity, and the other two quadrants displayed a far disparity. However, the disparity magnitude across all four windows was identical, such that the total disparity on a given frame was zero. Two main conditions were presented; one consisting of dot contrast that was fully correlated between the monocular images (correlated condition) and one where the dot contrast was fully reversed between the monocular images (anticorrelated condition). While the correlated condition led to a strong percept of depth, this was not the case for the anticorrelated stimulus. In both cases, binocular disparity was modulated with identical log-scaled disparity steps.

To control participant attention and alertness during the presentation, we introduced a contrast detection task. Each participant was instructed to maintain fixation and detect changes in the contrast of the random-dot stimulus and respond via an MRI-compatible button box. Contrast changes were introduced pseudorandomly 72 times during each run. Each event consisted of a 20% reduction in contrast, applied only to dots contained within the four disparity-modulated apertures. Contrast changes were ramped up and down to avoid a sudden change in contrast, potentially eliciting a blink response in the participant. Each change lasted 1 s, and they were spaced 5 s apart, with an additional ±2 s jitter. If the participant responded within 1 s of the contrast change onset, the event was classified as successfully detected. Participants detected an average of 76.3 ± 6.0% (SEM) of the contrast changes across all conditions.

#### Retinotopic mapping

Three participants took part in a previous study (Alvarez et al., 2021), and existing retinotopic delineations were used for those participants. The remaining seven participants underwent an additional scanning session for retinotopic mapping using stimuli identical to that used in the previous study. A full-field checkerboard stimulus, alternating with a gray background (2.5 s ON, 30 s OFF), was used to estimate the hemodynamic response function (HRF) of visual cortical responses in each participant. Next, a contrast-reversing radial checkerboard was presented through wedge and ring apertures to model the retinotopic organization of the visual cortex (Engel et al., 1994; Sereno et al., 1995; DeYoe et al., 1996). Four conditions were presented, clockwise rotating wedge, anticlockwise rotating wedge, radially expanding ring, or radially contracting ring. In each run, eight revolutions of the traversing aperture were presented, lasting 45 s each, with two periods of no stimulation lasting 30 s each. The total acquisition time for each run was 420 s. The resulting BOLD signals were fitted with a 2D Gaussian pRF model (Dumoulin and Wandell, 2008; Wandell and Winawer, 2015) using the participant-specific HRF in the model prediction. The polar angle and eccentricity estimates were used to delineate retinotopic boundaries for regions of interest V1, V2, V3, V4, VOC, V3A/B, V7, LOC, and V5/hMT+ (Silver and Kastner, 2009). A fixation task was presented throughout the run, where the participant responded to a brief (200 ms) change in the fixation point color occurring pseudorandomly 80–100 times each run. Responses were monitored to ensure participant alertness (events detected M = 98.04 ± 0.33% SEM).

#### MRI acquisition

MR images were acquired on a 3 T Prisma MRI system (Siemens Healthineers) using a 64-channel head coil (Siemens Healthineers). Functional images were acquired with a gradient echo EPI sequence (TR, 1,355 ms; TE, 32.40 ms; flip angle, 70°; 72 slices; resolution, 2 mm isotropic) with parallel multiband acceleration (MB factor, 4; Moeller et al., 2010). For the main task, four runs were acquired for each of the correlated and anticorrelated stimulus conditions, with 268 volumes acquired per run. The order of conditions was randomized across participants and acquisition split over two sessions, each lasting ∼1.5 h. Participants who performed the retinotopic mapping task underwent an additional scanning session, comprising four runs of 308 volumes with the same sequence.

B_0_ field maps were acquired in-plane in each run to estimate and correct distortions due to field inhomogeneity (TR, 482 ms; TE_1_, 4.92 ms; TE_2_, 7.38 ms; resolution, 2 mm isotropic). Finally, a T1-weighted (T1w) whole–brain anatomical image was acquired to reconstruct the cortical surface and anatomically localize functional data (MP-RAGE; TR, 1,900 ms; TE, 3.97 ms; TI, 904 ms; flip angle, 8°; slices, 192; resolution, 1 mm isotropic).

Eye tracking was not performed because the position of the stereoscope on the MRI head coil obscured the line of sight from the eye tracker to the eye.

#### Disparity tuning curve modeling

First, BOLD images were preprocessed with FSL (FMRIB Software Library v5.0.8; http://www.fmrib.ox.ac.uk/fsl). Images were brain-extracted, corrected for participant motion by linear realignment to the middle time point of each run, and temporally filtered to retain signals between 0.02 and 0.2 Hz (Jenkinson and Smith, 2001; Jenkinson et al., 2002; Smith, 2002). Each run was then registered to the participant-specific T1w structural image using boundary-based registration while simultaneously performing spatial correction for magnetic field inhomogeneities, as estimated by B_0_ fieldmaps (Greve and Fischl, 2009). Resulting BOLD signals were projected onto cortical surfaces reconstructed from T1w structural images in FreeSurfer (v6.0.0, http://www.freesurfer.net; Dale et al., 1999; Fischl et al., 1999; Fischl, 2012).

**Figure 2.**
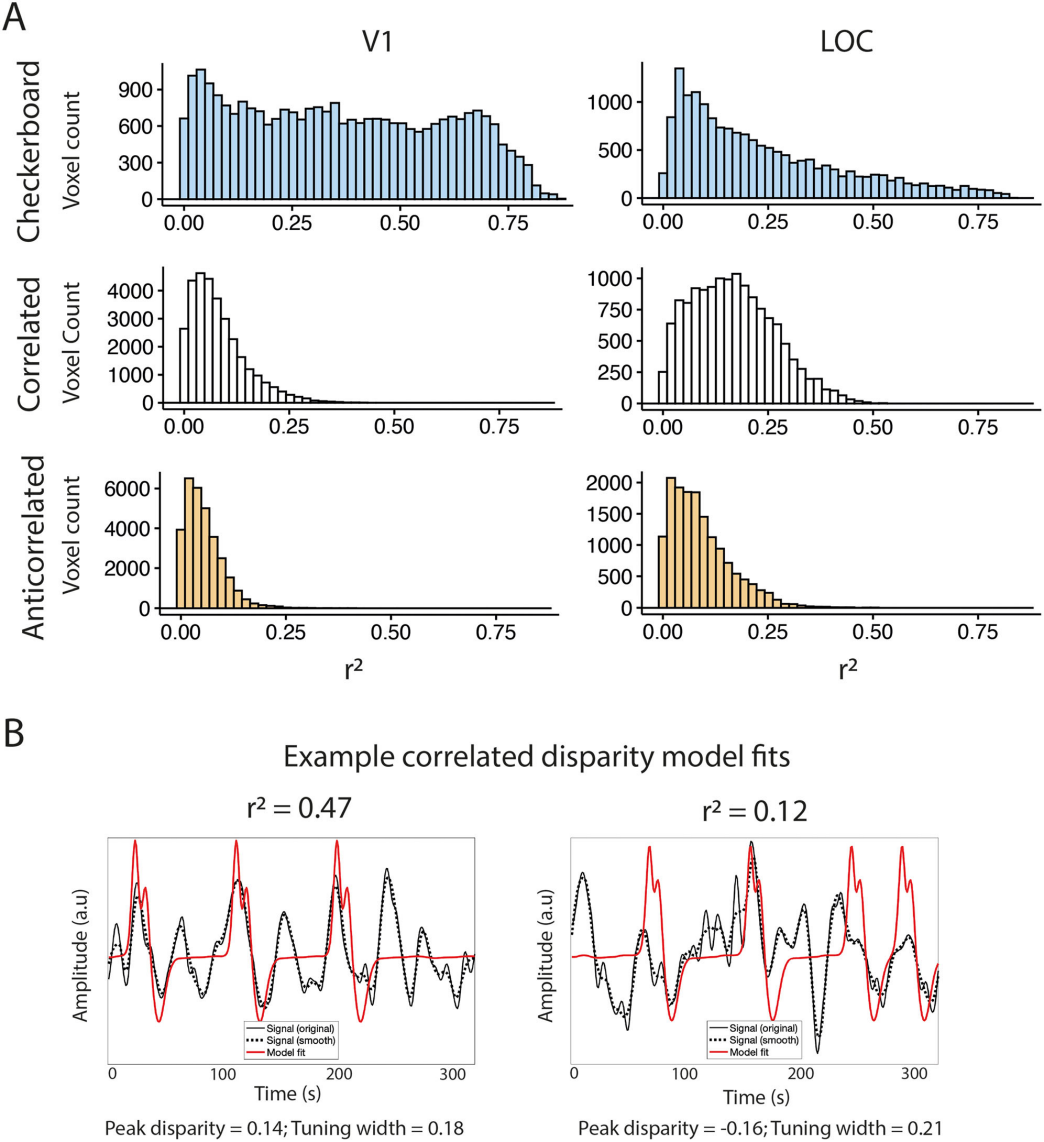
The distribution of *r*^2^ values in areas V1 and LOC across participants for the checkerboard data and the correlated and anticorrelated disparity data (***A***). It is immediately clear that the fits for the checkerboard data are better with some *r*^2^ values over 0.75. In comparison, the maximum fit for the disparity stimulus was ∼0.5 in LOC and <0.3 in V1. To illustrate the quality of fit for the correlated disparity, ***B*** shows example model fits with *r*^2^ values of 0.47 and 0.12.

Preprocessed BOLD signals were fitted with a one-dimensional Gaussian pRF model (Wandell and Winawer, 2015). The analysis software was implemented in MATLAB and has been described for a two-dimensional implementation (Alvarez et al., 2015). Model predictions for a given quadrant aperture were generated by combining the disparity values shown during stimulation and a Gaussian tuning curve, defined by three parameters: peak disparity (μ); width of the tuning curve, expressed in standard deviations (σ); and an amplitude parameter (β). The model prediction was convolved with the participant-specific HRF and compared with the observed signal in a two-stage procedure. First, BOLD signals were spatially smoothed (FWHM, 5 mm on spherical mesh) and compared with 10,000 model predictions generated with varying parameter values in an exhaustive grid search procedure. The parameter estimate that yielded the largest correlation was then used as the seed point for a nonlinear optimization procedure, where the parameter estimates are refined against the original BOLD signals. This procedure is carried out independently for each surface vertex and model performance assessed with the coefficient of determination (*r*^2^). Only vertices with model goodness-of-fit *r*^2^ > 0.1 were retained for further analysis. Figure 2*A* shows the distribution of *r* across areas V1 and LOC for all participants. For the checkerboard contrast stimulus, V1 shows a relatively flat distribution across a wide range of values. For the disparity stimulus, there are no values above 0.6, indicating that the 1D model fitted to these data is generally not as good as the 2D standard spatial model. Moreover, for V1, the number of voxels in each bin drops off steeply, and the distributions for correlated and anticorrelated stimuli are rather similar. In contrast, the values for LOC are higher on average and the correlated random-dot stimuli show considerably more values >0.25. Examples of data fits to correlated disparity stimuli at different *r*^2^ values are shown in Figure 2*B*. Given the relative noisiness of the disparity data compared with the checkerboard data, we opted for the relatively conservative threshold of 0.1 that has been used previously to identify the vertices with a reasonably robust fit in datasets with higher noise (Dekker et al., 2019).

Two tuning curve parameters were taken as outcome measures: peak disparity and tuning curve width. Peak disparity was defined as the disparity value, positive or negative, eliciting the maximum response. Tuning curve width was defined as ±2σ of the fitted Gaussian, approximately equivalent to the “disparity scale” defined for Gabor functions in Parker (2007). A lower limit of 0.1° was applied to the tuning curve width, reflecting the lowest values recorded for the disparity scale in the neurophysiological data from Prince et al. (Prince et al., 2002a,b). Values smaller than 0.1° were therefore considered to be nonphysiological and a result of model fitting error and hence excluded from further analysis. Table 1 shows the number of vertices for each visual area excluded by first setting the *r*^2^ > 0.1 threshold and then tuning width >0.1° for both the correlated and anticorrelated stimuli.

**Table 1.** The effect of thresholding on the number of vertices included in the analyses

	V1	V2	V3	V4	VOC	V3A/B	V5/hMT+	LOC	V7
Correlated vertices	31,383	26,056	18,655	7,007	7,040	9,161	11,055	14,012	5,233
Correlated vertices: *r*^2^ > 0.1	9,606	11,343	9,920	2,559	3,331	7,195	6,786	9,976	3,961
Correlated *r*^2^ > 0.1 and width > 0.1	8,463	9,895	8,849	2,131	2,978	6,754	6,333	9,412	3,776
Anticorrelated vertices	31,124	25,866	19,583	7,025	7,817	9,241	11,060	15,320	6,057
Anticorrelated vertices: *r*^2^ > 0.1	4,332	5,509	5,849	1,066	1,839	2,630	2,452	5,507	1,263
Anticorrelated *r*^2^ > 0.1 and width >0.1	3,796	4,178	3,947	733	1,338	1,729	2,170	3,419	915

Adding *r*^2^ threshold reduced the number of vertices considerably for both the correlated and anticorrelated conditions. Including the additional constraint that the tuning width needed to be >0.1° reduced the number of vertices by ∼10% across all visual areas.

Surface points inside a visual area were assigned a retinotopic quadrant based on the estimated pRF location, derived from the retinotopic mapping task. The disparity stimulation pattern for the corresponding quadrant was then used as the regressor when fitting the disparity tuning curve model for a given vertex. The correlated and anticorrelated stimulus conditions were fitted independently.

### Psychophysical stereoacuity

#### Stimulus presentation

Visual stimuli were generated in MATLAB using Psychtoolbox and delivered through a custom Wheatstone stereoscope comprised of two CRT monitors (ViewSonic E70fSB 17″; resolution, 1,024 × 768; display area, 31.82 × 24.26°; viewing distance, 570 mm) viewed through cold mirrors. The participant sat at a head and chin rest, with blinkers placed at the temporal aspect of the head to avoid direct observation of the monitors. Eye position was monitored with an infrared eye tracker (EyeLink 1000 Plus, SR Research) and analyzed with the EyeLink Toolbox (Cornelissen et al., 2002).

#### Experimental design

**Figure 3.**
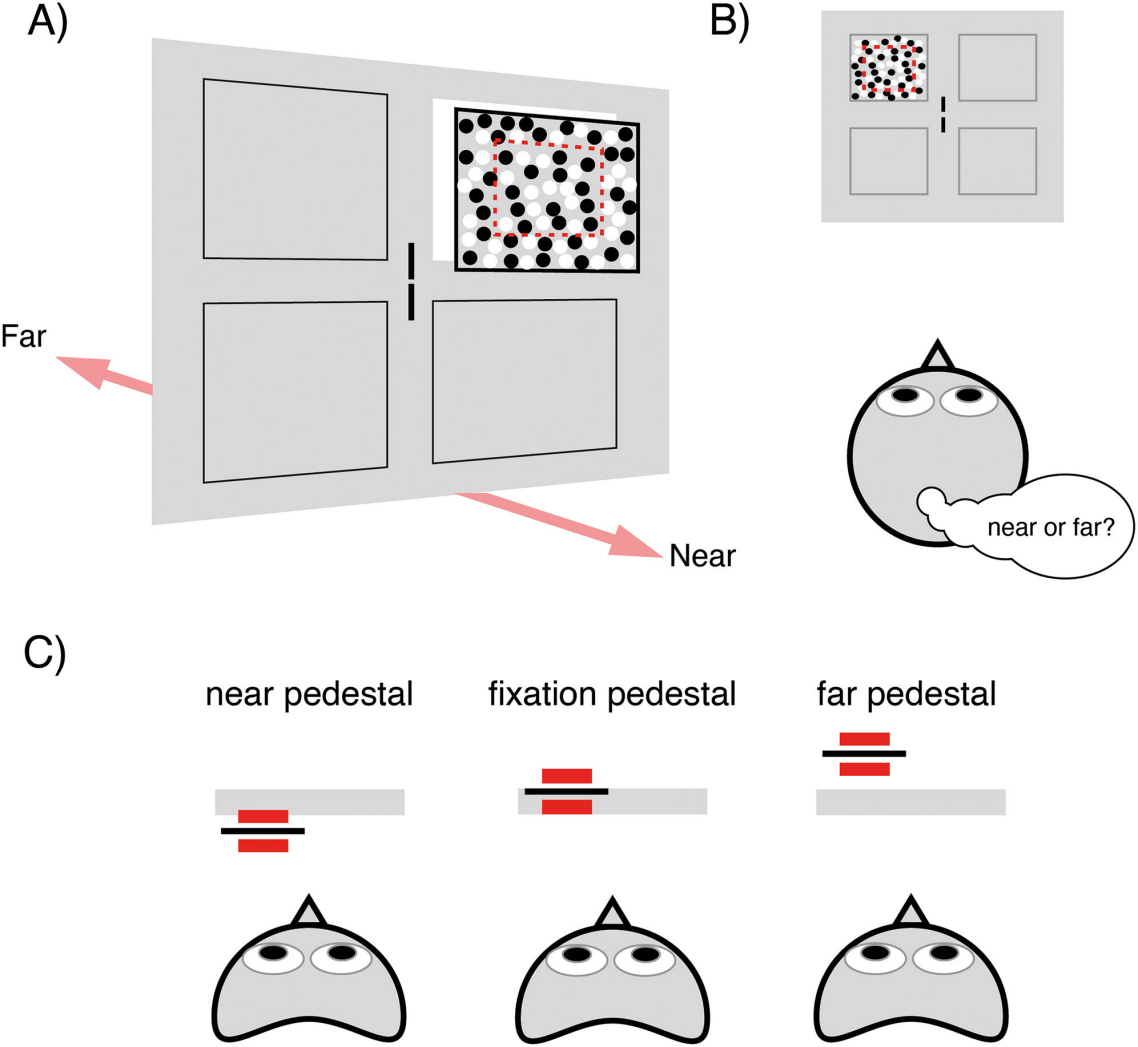
Task design for measuring psychophysical stereoacuity. ***A***, The stimulus display consisted of a blank screen with zero-disparity nonius lines at fixation and a single RDS at one of the four possible quadrant positions (indicated by the black outlines). The stereogram (7 × 7°) was composed of two parts—a surround pedestal with fixed disparity and a 4 × 4° target defined by relative disparity (dotted red outline). ***B***, While maintaining fixation, the participant observed the stereogram for 1,000 ms and, in a 2AFC task, decided whether the relative disparity target was near or far in relation to the pedestal disparity. ***C***, Three pedestal disparities were tested, zero disparity (fixation), a near pedestal (−0.4°), and a far pedestal (+0.4°). The disparity in the target zone varied adaptively relative to the pedestal disparity depending on the participant response.

The psychophysical testing was designed to measure stereoacuity at the locations in the visual field that corresponded to the quadrants in which the fMRI stimuli for mapping depth pRFs were located. In addition, stereoacuity was measured relative to backgrounds at different locations in depth with respect to the fixation plane; “pedestal disparities” as well as around zero disparity. Again, this was to permit comparison of stereoacuity thresholds with the tuning width of depth pRFs, since the tuning width is likely to vary with preferred disparity. For example, stereoacuity thresholds at a “far” pedestal disparity can be compared with the tuning width of vertices that have a “far” preferred disparity. The experimental task was a 2AFC design in which participants rated a disparity-defined surface as being near or far relative to the disparity pedestal background (Fig. 3).

The stimulus consisted of a pair of zero-disparity nonius lines (width, 0.1°; height, 0.8°) in the center of the display as a cue for fixation and a 7 × 7° RDS consisting of 1,000 correlated black and white dots (dot radius, 0.05°; refresh rate, 30  Hz; 100% contrast) presented in one of four cardinal quadrants. The quadrants were equally spaced from the fixation point, with the RDS center positioned 5.5° eccentric from fixation. Within the RDS, a pedestal disparity was set to one of three values (0°, +0.4°, and −0.4°), and a central 4 × 4° aperture was defined by relative disparity against the pedestal. In a single trial, the participant was required to align the nonius lines, the RDS appeared in a randomized quadrant location for 1,000 ms, and the participant responded after the RDS presentation fully elapsed. Participants were instructed to report the relative depth direction (near vs far) of the target aperture as fast and as accurately as possible while maintaining fixation. Audio feedback was given after every trial to indicate if a response was correct or incorrect. If, during RDS presentation, horizontal gaze position deviated by >1.80° from fixation, the trial was rejected, and the participant viewed a screen stating, “please fixate.”

Stereoacuity thresholds were estimated with a two-stage procedure, using log-spaced disparity steps. First, a two-down, one-up staircase was used to generate initial convergence (Wetherill and Levitt, 1965). Starting at 0.1° relative disparity, participants performed trials until three reversals in the psychometric response were observed with a step size of 0.5 log decades and then a further three reversals with a step size of 0.1 log decades. The disparity at the final reversal point was used as the initial value in the following stage. Next, a dual staircase adaptive procedure implemented in QUEST was used (Watson and Pelli, 1983; Watson, 2017). For each condition, half the trials were randomly assigned to be sampled at the 30% threshold point of the adaptive Weibull function and half the trials to be sampled at the 70% threshold point. This ensured wide sampling of the slope of the psychometric function.

In total, 12 conditions were tested by combining four retinotopic quadrant locations (upper left, upper right, lower left, lower right) and three pedestal disparities (fixation, near, far). Each condition had a separate staircase, and trials were randomized across conditions throughout the experiment. Participants completed 50 trials per condition in the dual staircase procedure, for a total of 600 trials during the second stage. The number of trials during the convergence stage was variable and averaged 16.72 (±3.74 SD) trials across participants. The percentage of trials rejected due to poor fixation behavior averaged 13.80% (±15.10% SD) across participants. Rejected trials were discarded, to ensure all participants completed 600 successful trials during the second estimation stage. The posterior QUEST estimate for psychophysical stereoacuity for each condition was then taken as the outcome variable.

### Statistical analysis

Statistics were performed in GraphPad Prism. A two-way ANOVA was performed to determine whether the proportion of vertices differed across visual areas and stimulus conditions (correlated and anticorrelated).

To determine the optimal fit of the tuning curve width and disparity preference, we performed a least-square regression of tuning width against preferred disparity, first using a straight line and also using a second-order polynomial constrained to have a minimum at *x* = 0. Since both fits have the same number of degrees of freedom, Akaike's information criterion (AICc) was used to determine the better fit.

Psychophysical thresholds were analyzed with a two-way ANOVA, with stereoacuity threshold as the dependent variable, visual field position, and disparity pedestal as the within-subject independent variables and subject identity as the between-subject independent variable.

## Results

### Disparity sensitivity across the visual cortex

**Figure 4.**
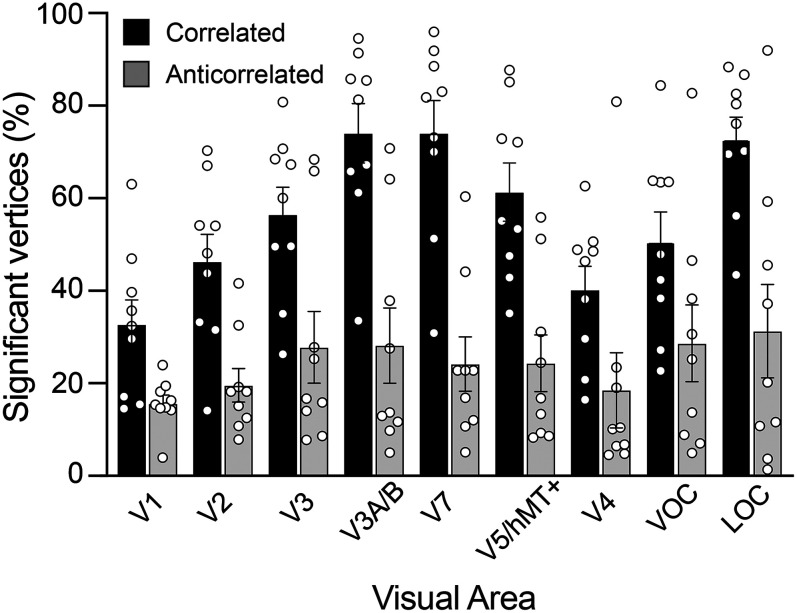
The percentage of vertices in visual cortical areas where BOLD responses are significantly explained by the disparity tuning curve model (*R*^2^ > 0.1). Independent model fits under correlated and anticorrelated disparity modulation are shown. Vertices were selected for further analysis if (1) their spatial receptive fields, as estimated with retinotopic mapping, were located within the visual field zone stimulated by disparity changes and (2) responses to correlated RDS stimulation survived goodness-of-fit thresholding at *R*^2^ > 0.1. The baseline for both conditions was all vertices activated by the correlated stimulus (*R*^2^ > 0). Bars show mean ± standard error across participants and circles show individual participant percentages (*n* = 9).

Sensitivity to disparity information was found across the visual cortex, with up to 95% of vertices within a visual area responding significantly to correlated RDS stimulation across the visual areas tested in individual participants (Fig. 4). One participant was excluded from all analyses because over 85% of fitted vertices showed a preferred disparity clustered around the values of +0.23 or −0.23° in V1. If fitting is working correctly, there should be a range of preferred disparities in V1. One reason for peaks at these values could be vergence eye movements away from the zero-disparity plane, when the stimuli reached maximum disparity, or there was a failure of fitting, due to poor data quality. The percentage of significant vertices was calculated by first quantifying the vertices within each visual field zone stimulated by disparity changes that had good model fit *r*^2^ > 0.1 (for both correlated and anticorrelated stimuli). This was then compared with the number of vertices in same region but that had *r*^2^ ≥ 0 for the correlated stimulus.

A two-way repeated–measure ANOVA with main effects of visual area and stimulus type (correlated or anticorrelated) showed that the number of vertices in each visual area responsive to disparity information varied significantly (two-way ANOVA; *F*_(8,72)_ = 4.5; *p* = 0.0002). For correlated disparity, the highest proportion of cortical territory responsive to disparity information was found in the dorsal and lateral visual cortex, specifically areas V3A/B, V7, and LOC. This is consistent with the known specialized role for disparity processing (Backus et al., 2001; Tsao et al., 2003; Preston et al., 2008). A significantly smaller proportion of the cortical vertices responded significantly to anticorrelated RDS stimulation (two-way ANOVA; *F*_(1,72)_ = 110; *p* < 0.0001). However, there was no interaction between the stimulus type and visual area (*F*_(8,72)_ = 1.6; *p* = 0.13). Nonetheless, the proportion of vertices selective for anticorrelated stimuli was relatively consistent across areas, whereas there was a large proportion of vertices selective for correlated disparity in dorsal and lateral areas. We designated the subset of vertices with significant responses to correlated RDS as “disparity-sensitive” vertices and subjected them to further analysis to assess their disparity tuning properties.

#### Distribution of preferred disparities varies across the visual cortex

**Figure 5.**
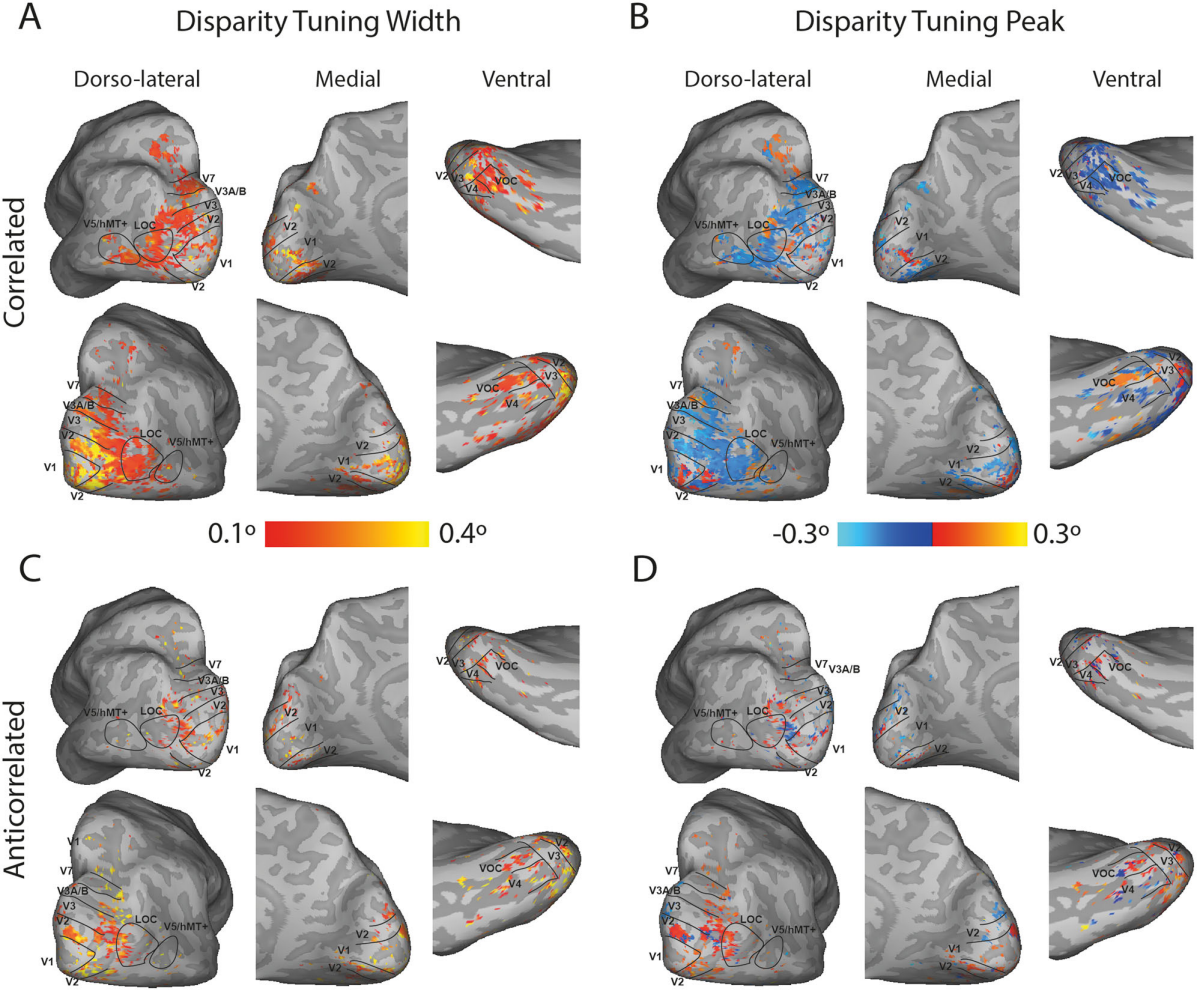
***A***, The disparity tuning width of each of the vertices tuned to correlated binocular disparity on the inflated cortical surface of an example participant (D001). ***B***, The preferred disparity of each of these vertices. ***C***, ***D***, The disparity tuning width and preferred disparity, respectively, for the vertices responding to anticorrelated disparity. Vertices are thresholded at *R*^2^ > 0.1 and disparity tuning width >0.1°. Note that tuning widths are not thresholded at 0.4°, but any greater values would appear yellow. Visual areas from retinotopic mapping are superimposed.

The topographic distribution of binocular disparity tuning curve properties revealed two trends. First, cortical visual areas contained a range of peak preferred disparities, and second, as predicted by Figure 4, responses to anticorrelated RDS stimulation were considerably more limited, both in spatial extent and in the range of disparity tuning curve profiles exhibited. Figure 5 shows example data from one of the participants, reflecting the large swathe of the dorsal and lateral occipital lobes and intraparietal sulcus that responded significantly to correlated disparity-defined stimuli. Specifically, it is evident that the entire range of disparity values is represented in this region (Fig. 5*B*), as may be seen from the colors representing near and far disparity values. Similarly, the variability of disparity tuning width is also visible across this region (Fig. 5*A*). The comparable data from the anticorrelated disparity condition are considerably more sparse (Fig. 5*C*,*D*).

**Figure 6.**
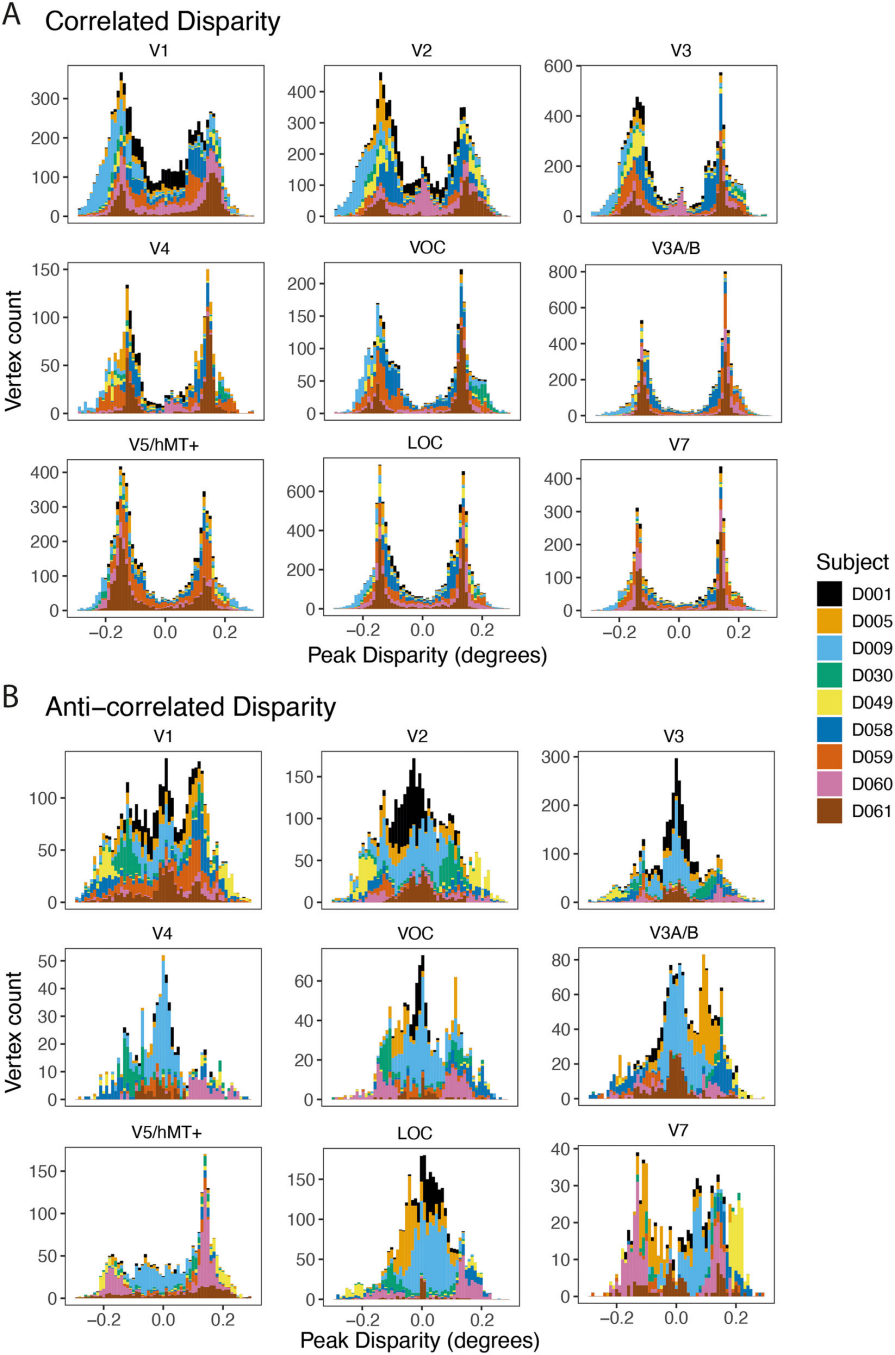
Distribution of disparity-sensitive cortical vertices across peak disparities in nine visual areas across participants. ***A***, The counts of vertices responsive to correlated RDS stimulation divided by participant (total *n* = 58,591). ***B***, The counts of vertices responsive to anticorrelated RDS stimulation (total *n* = 22,206). Note that the axes are different across the visual areas to better illustrate the distribution of preferred disparity according to participant. The preferred disparities in the correlated condition peak ∼ 0.15°, particularly in higher visual areas, both dorsal and ventral. In contrast, in the anticorrelated condition, the preferred disparities are centered close to zero, where present.

To determine the representation of preferred disparity across the range tested, we quantified, for each participant, the number of vertices in each retinotopically defined visual area with a peak value lying within each of 50 disparity bins. Figure 6 shows these distributions for each individual visual area, color-coded for each of the nine participants who showed significant activation. Figure 6*A* shows the distributions for the correlated stimuli, with vertices selective for a range of peak disparity values across those tested. Consistent with Figure 4, there was considerable variability in the number of vertices activated across participants, although no single participant significantly dominated the distribution. The number of activated vertices was considerably lower for the anticorrelated (B) compared with the correlated stimuli.

The distribution of preferred disparity varied significantly across visual areas, particularly for the correlated disparity condition. V1 and V2 have similar distributions, with peaks around ±0.15° but also a considerable number of vertices selective for values ∼ 0°. In higher visual areas, the majority of vertices across participants have a preference for nonzero disparity. While one participant (D061) shows this pattern to the greatest extent, it is also the case for the majority of participants.

In contrast to the responses to the correlated stimulus, the responses to the anticorrelated stimulus, while smaller, have a more even distribution of peak disparity. V1 clearly contains vertices tuned to many different disparities, with all participants contributing to the distribution. In higher visual areas, the responses are dominated by a subset of participants, with the rest showing few active vertices.

#### Disparity tuning curve width increases with peak disparity magnitude

**Figure 7.**
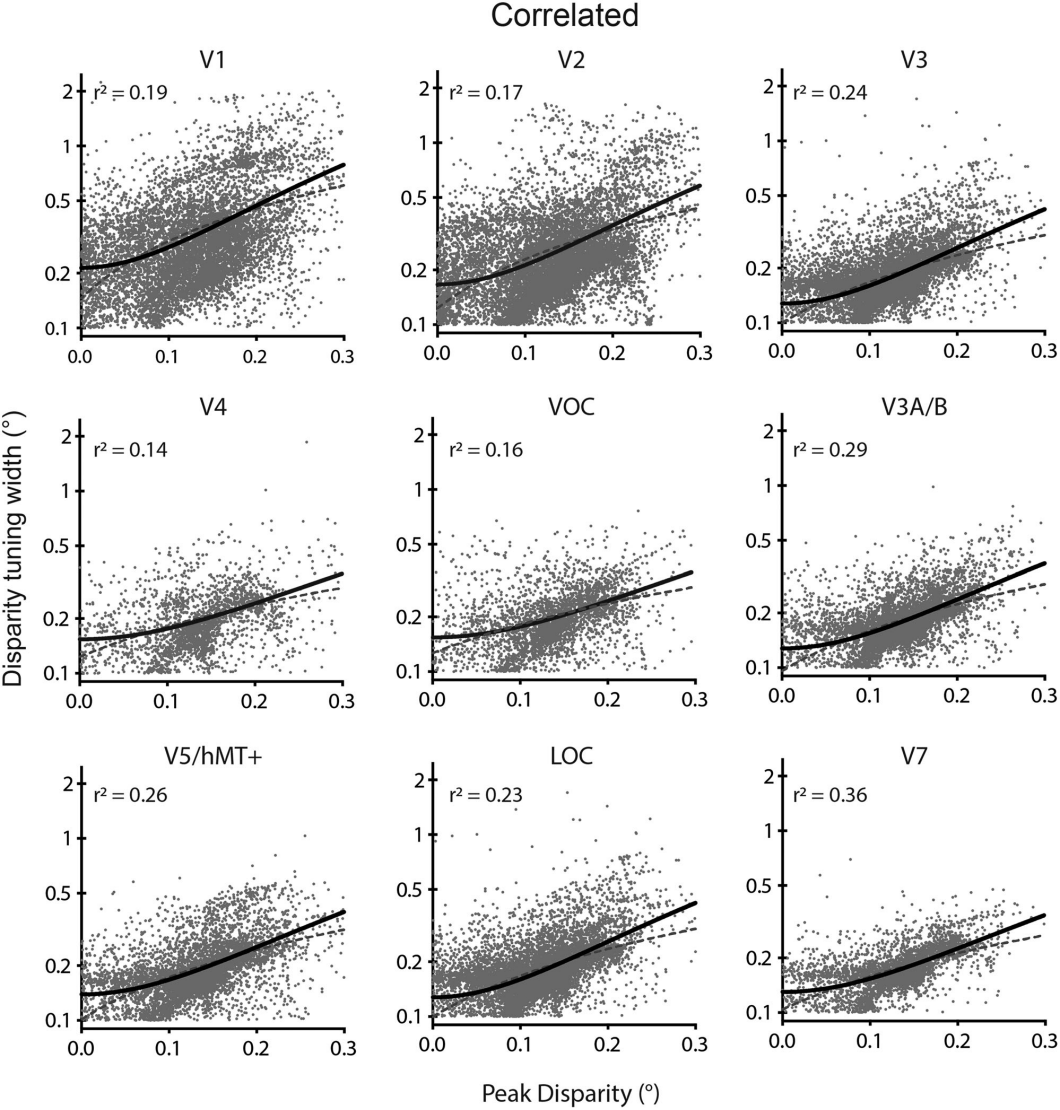
Relationship between the modulus of peak binocular disparity and disparity tuning curve width in response to correlated disparity across visual areas. In each case, the polynomial fit was superior to a straight line. Point cloud shows all vertices significantly modulated by correlated RDS stimuli across participants. The black line shows the best-fitting second–degree polynomial curve, and the gray dashed line is the straight line fit. Goodness of fit for the polynomial is quantified by a least-squares regression. Note that 0.1° was the lower limit for the width of the tuning curve and values below that were excluded.

The disparity tuning width from the model fit reflects the range of binocular disparities to which the specific vertex is responsive. As individual neurons tuned to large disparities tend to have larger receptive fields than those tuned to smaller disparities (Nienborg et al., 2004) and disparity-sensitive neurons with large receptive fields exhibit wider tuning curves (Prince et al., 2002a), we may reasonably expect a relationship between the modulus of the preferred disparity and tuning curve width to hold at the cell population level as probed with fMRI techniques. Assessing the population tuning curve estimates for correlated disparity, using a least-squares regression of width against preferred disparity, revealed that a second-order polynomial was required to describe the relationship in all visual areas (Fig. 7). In each case, the polynomial was constrained to have a minimum at *x* = 0, such that it had the same number of parameters as a straight line. Dorsal regions V3A/B and V7 showed the best fit to the polynomial, while the relationship was weakest in ventral areas V4 and VOC. Nonetheless, when the polynomial fit was compared against a straight line fit using AICc, the polynomial was a significantly better fit in all areas [V1, difference in AICc (ΔAICc) = 349; V2, ΔAICc = 596; V3, ΔAICc = 870; V4, ΔAICc = 59; VOC, ΔAICc = 137; V3A/B, ΔAICc = 831; V5/hMT+, ΔAICc = 392; LOC, ΔAICc = 1,176; V7, ΔAICc = 639; probability that polynomial is a better choice is >99.99% in all cases].

**Figure 8.**
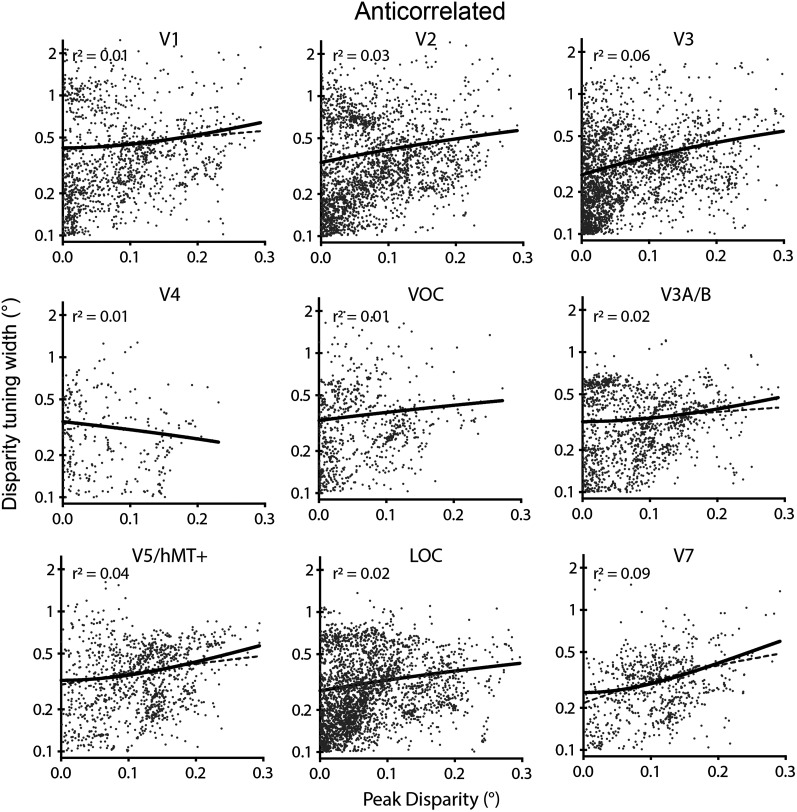
Relationship between the modulus of peak binocular disparity and disparity tuning curve width in response to anticorrelated disparity. The polynomial fit is only superior to a straight line in LOC. Point cloud shows all vertices significantly modulated by anticorrelated RDS stimuli across participants and has considerably fewer points than in Figure 7. The black line shows the best-fitting second–degree polynomial curve (centered on *x* = 0), and the gray dashed line is the straight line fit. Goodness of fit for the polynomial is quantified by a least-squares regression. Note that 0.1° was the lower limit for the width of the tuning curve.

The relationship between the modulus of preferred disparity and tuning curve in response to anticorrelated stimuli was also fit with a second-order polynomial centered at *x* = 0 and compared with a line (Fig. 8). In each case, the number of vertices that were included was lower than in the correlated case, consistent with the data shown in Figures 4–6. Since the tuning width is defined as ±2σ, it is possible for the width to be greater than the range of disparities presented. Only in area LOC was the polynomial fit significantly better than a line (probability > 99.99%; LOC, ΔAICc = 30). The remaining areas had probability <99.99% for the polynomial fit (V1, ΔAICc = 4; V2, ΔAICc = 3; V4, ΔAICc = 1; VOC, ΔAICc = 3; V7, ΔAICc = 6), and V3 showed better fit for the line (V3, ΔAICc = −26).

### Relating psychophysical stereoacuity to disparity tuning curves

#### Worse stereoacuity when judging depth in the “far” depth plane

**Figure 9.**
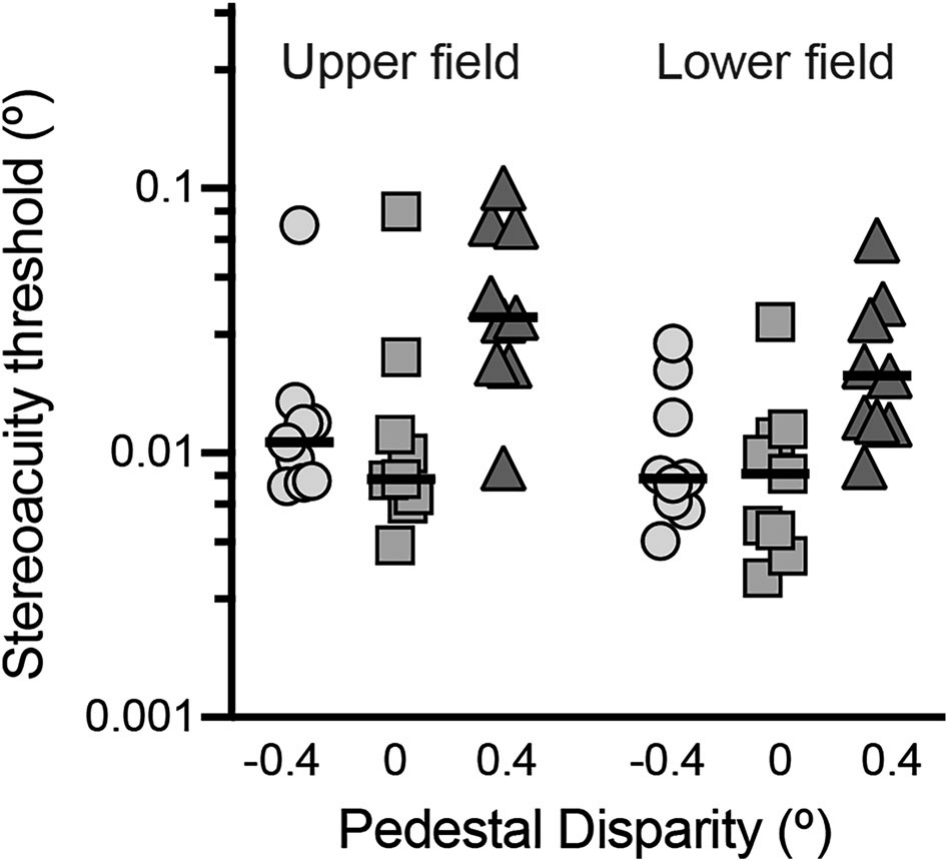
Stereoacuity thresholds for detecting disparity relative to near (−0.4°, circles), fixation (0°, squares), and far (+0.4°, triangles) disparity pedestal positions. There was no difference between the upper and lower fields, but the “far pedestal” had significantly higher thresholds than fixation and “near pedestal” in both upper and lower fields. Performance for individual participants is shown by the symbols, with the median indicated by the black horizontal line.

Relative disparity stereoacuity was assessed at four eccentric visual field locations, across three disparity pedestals; fixation (0°), near (−0.4°), and far (+0.4°). As no difference was expected between left and right visual fields, estimates were collapsed across the vertical meridian to obtain estimates for the upper and lower visual fields (Fig. 9). Thresholds were analyzed with a two-way ANOVA, with stereoacuity threshold as the dependent variable, visual field position, and disparity pedestal as the within-subject independent variables and subject identity as the between-subject independent variable. A significant main effect of disparity pedestal was detected (*F*_(1.1, 17.7)_ = 27.9; *p* < 0.0001), with no significant main effect of visual field position (*F*_(1,17)_ = 1.4; *p* = 0.21) or overall interaction (*F*_(2,32)_ = 2.8; *p* = 0.07). Post hoc Šídák tests revealed that stereoacuity thresholds were worse with a far pedestal, compared with no pedestal (upper hemifield, *p* = 0.009; lower hemifield, *p* = 0.01) and to the near pedestal (upper hemifield, *p* = 0.01; lower hemifield, *p* = 0.03). Thus, while there was an effect of adding a stimulus pedestal, this was driven predominantly by an increase in stereoacuity thresholds with a far disparity pedestal.

#### Neural correlates of psychophysical stereoacuity

**Figure 10.**
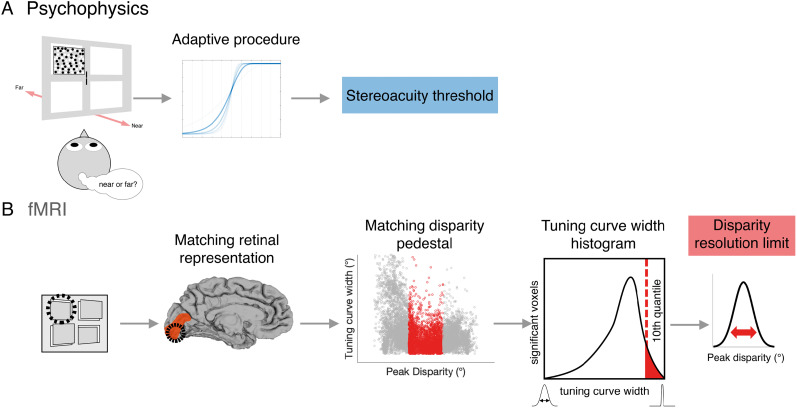
Quantitative inputs for relating psychophysical stereoacuity thresholds and equivalent disparity pRF tuning widths. ***A***, In the psychophysical assessment of stereoacuity, stimuli were presented in four visual field locations, at three possible disparity pedestals. Each participant's responses to a 2AFC task were fitted with an adaptive procedure, and stereoacuity thresholds obtained for each condition tested. ***B***, For each visual field location in the fMRI experiment, the matching cortical representation in each visual area was selected based on retinotopic mapping of spatial receptive field location. Next, the disparity tuning curve model fits obtained under correlated RDS stimulation were extracted and divided into disparity bins selected to match the disparity pedestals used in the psychophysical task. Finally, for a given bin, the 10% quantile was calculated, and the value was taken as the disparity resolution limit, reflecting the sharpest population-level disparity tuning curves observed at the sampled cortical location for each participant.

**Figure 11.**
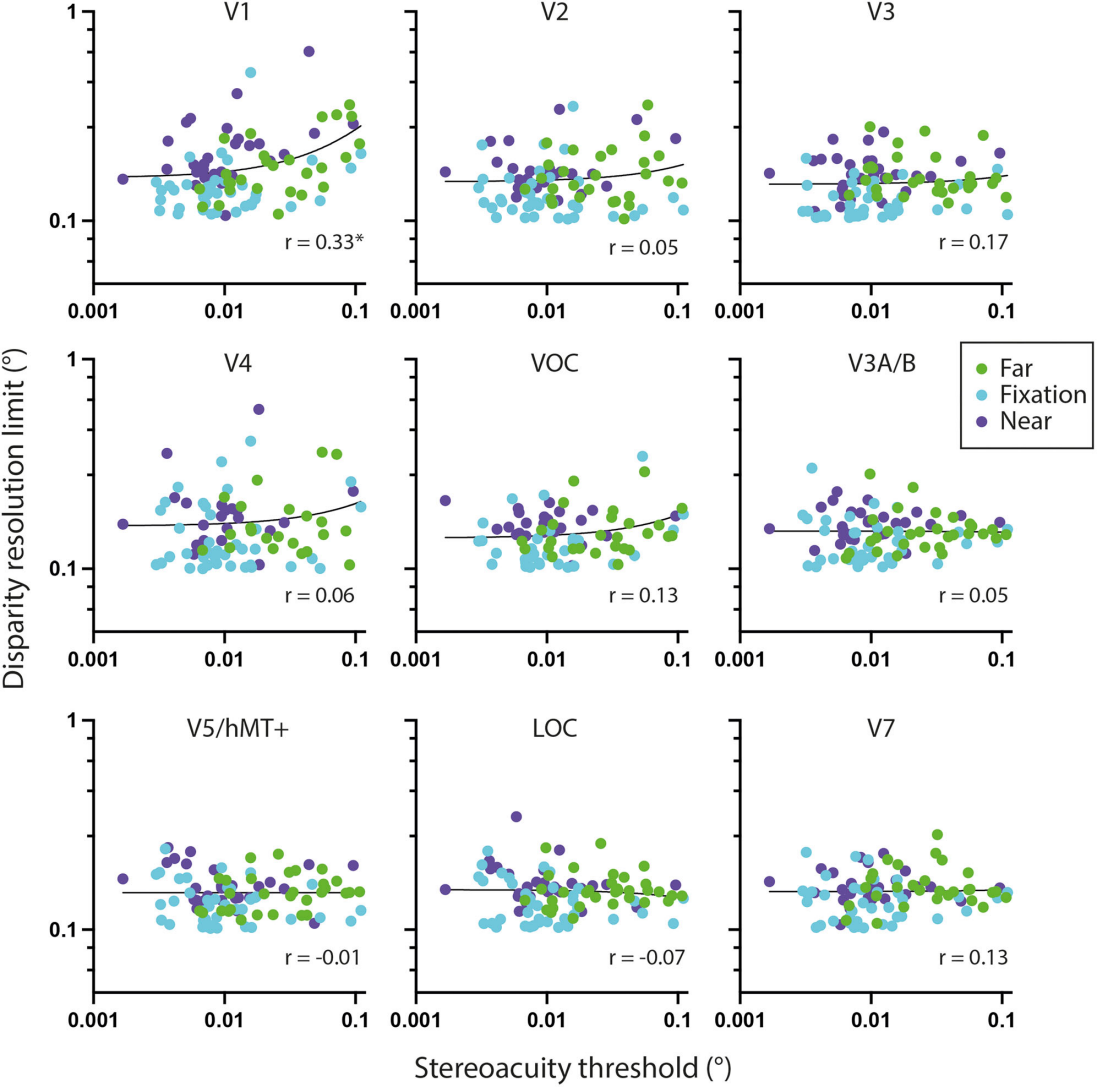
Relationship between psychophysical stereoacuity and the resolution limit of pRF disparity tuning curve widths. Each point corresponds to a psychophysical stereoacuity estimated at a given visual field location (upper or lower visual field), hemifield (left or right) at a binocular disparity pedestal (far, green; fixation, cyan; near, purple) in a single participant, yielding 12 data points per participant. The disparity resolution limit corresponds to the 10% quantile value for the matched pRF tuning curve widths (see text for details). The linear regression line is shown in black. Spearman's correlation coefficients shown in text inserts, with only V1 displaying a significant correlation after correcting for nine comparisons.

We compared psychophysical stereoacuity across the visual field at the three disparity pedestals for each participant with disparity pRF tuning curve width at the same locations in depth, i.e., with the preferred disparity near, far, or around zero. Specifically, stereoacuity thresholds were obtained for each participant, in each visual hemifield (UVF, LVF), at each disparity pedestal tested (fixation, near, far). Next, for each of these locations, a subset of disparity-sensitive vertices was selected, so that (1) they matched the visual field location tested in their retinotopic pRF location and (2) the peak disparity under correlated RDS broadly matched the tested pedestal, since the range of disparities were between ±0.3°. Specifically, vertices with peak disparities less than −0.1° were assigned to the “near” pedestal (compared with −0.4° pedestal), those with peak disparities between −0.1 and +0.1° assigned to the “fixation” pedestal and those with peak disparities greater than +0.1° assigned to the “far” pedestal. Thus, all disparity selective vertices were used but binned into the different disparity pedestals and into upper and lower visual fields. For each subset of vertices, the 10% quantile value was used as a measure of the smallest average disparity this region can encode. We selected this metric to use as the “resolution limit” of the population disparity tuning curve (Fig. 10). This disparity resolution limit was then correlated with the matched stereoacuity threshold for each participant, across all psychophysical task conditions, as shown in Figure 11. A significant correlation between the pRF disparity resolution limit and stereoacuity threshold was only found for area V1 (*r* = 0.33; *p* = 0.008; *n* = 95; Bonferroni corrected for nine visual areas). Correlations in all other visual regions were nonsignificant. The number of points in each case should be 36 per pedestal value, with a total of 108. However, some participants did not have any significant vertices at the relevant spatial location and depth. Table 2 shows the number of missing points for each visual area and pedestal disparity.

**Table 2.** The number of missing data points for each condition in Figure 10

	V1	V2	V3	V4	VOC	V3A/B	V5/hMT+	LOC	V7
Near	4	5	3	11	8	2	2	2	3
Fixation	1	1	1	2	6	1	1	0	1
Far	8	9	6	14	9	7	6	3	7

There should be 36 points per cell. In every case, the largest number of missing values is for the vertices tuned to “far” (average 7.6/36) and the lowest for those around fixation (average 1.5/36). Ventral regions V4 and VOC have the largest number of missing points as might be predicted from Figure 5.

To ensure that the use of the 10th quantile did not bias the results, we performed the same correlation using the median value. These results were consistent with a significant correlation between pRF median tuning width and stereoacuity threshold for V1 only (*r* = 0.31; *p* = 0.02; *n* = 95; Bonferroni corrected for nine visual areas).

## Discussion

Using pRF mapping, we have shown that it is possible to describe the properties of binocular disparity tuning curves estimated in vivo in humans across retinotopically defined visual areas. The findings were in general agreement with a heterogeneous distribution of disparity tuning across visual areas. Near and far disparities were represented across the visual areas sampled, with responses to correlated RDS stimuli showing a wide range of preferred disparities and significantly lower responses to anticorrelated stimuli. Notably, there was a significant correlation between preferred disparity and disparity tuning width.

### Disparity selectivity is present across the visual cortex

A distinct advantage of fMRI approaches is the ability to simultaneously quantify neural responses across multiple brain areas. This presents the opportunity to sample the range of disparity selectivity contained within each visual area. With this in mind, we describe three main findings: (1) more vertices showed disparity tuning to correlated, compared with anticorrelated, stimuli, although a proportion of vertices did show disparity tuning to anticorrelated stimuli, (2) tuning curve width is related to preferred disparity such that vertices tuned to zero disparity have the narrowest tuning, and (3) the tuning width of vertices in V1 is correlated with stereoacuity in comparable regions of the visual field.

### Peak disparity extracted from the pRF model is biased toward values away from zero, particularly in higher visual areas

When the disparity stimulus was correlated, there was a clear bias in the number of fitted vertices to values around **±**0.15**°** in a number of the participants. This type of nonuniform distribution would not be predicted from the neurophysiological literature, where there is an ordered progression of disparity preference in some areas including V5/MT (DeAngelis and Newsome, 1999). While V1 and V2 showed a reasonable number of vertices tuned to disparities around zero, this was not the case for higher areas such as V3A/B and LOC. The peaks around **±**0.15**°** are likely the point at which the separation between the zero-disparity background and plane moving in depth becomes easily visible. Thus, in higher visual areas, where figure-ground segregation resulting from relative disparity is a potent stimulus (Cottereau et al., 2011; Kohler et al., 2019), the relative disparity between the zero-disparity background and stimulus appears to preferentially drive the neural responses. In addition to the **“**low level” figure-ground disparity responses, the BOLD signal has been shown to vary according to feature attention (Foster and Ling, 2022) which could also increase the preference for these nonzero values.

An additional complication is that the disparity pRFs defined in this manuscript measure the BOLD response to a change in disparity. However, the size of spatial receptive fields is likely to interact with this selectivity. This is particularly the case in higher visual areas where receptive fields tend to be larger, particularly at greater eccentricities, and will therefore include larger regions of the stimuli, often including stimulus background.

### Direct comparison of disparity pRF tuning width to neurophysiology is not straightforward

The range of tuning width that can be measured depends primarily on the specific disparities used in the stimuli. In the current study, it was important that participants could fuse the stimuli, as a loss of fusion leads to a rapid drop in fMRI signal and could affect fusion even when the stimulus moved back within the fusional range. While larger disparities can drive vergence (Georgeson and Wallis, 2014), the aim here was to avoid vergence as it was important that the disparity in each quadrant of the stimulus modulated ∼ 0° disparity, i.e., the fixation plane. Thus, the range of disparities explored was considerably narrower than is commonly used in neurophysiology where disparities up to (and beyond) ±1° are common (Prince et al., 2002a).

In addition to the reduced range of disparity, a second challenge for comparison with neurophysiological data is the definition of tuning width. The values used in the current study are related to the standard deviation (σ) of the Gaussian that is fitted to the fMRI data. The standard deviation relates to the full-width at half-height with a factor of 2.35. The neurophysiologically recorded disparity tuning curves are generally fitted with a Gabor function (the product of a Gaussian envelope and a sinewave). Extracting comparable measures is therefore not straightforward. The “disparity scale” that is used to describe the neurophysiology data in Parker (2007) is simply the reciprocal of disparity frequency in Cumming and DeAngelis, specifically their Figure 8 (Cumming and DeAngelis, 2001; Parker, 2007). Disparity frequency is “the peak frequency in the continuous Fourier transform of the disparity tuning curve.” If the Gabor model is used to describe a disparity tuning curve, then the values represented by “disparity scale” are actually the width of an excitatory region, together with its neighboring flank. Thus, on average, disparity scale derived in this way is twice the width of a single excitatory region in the receptive field. Modeling the same regions with a Gaussian tuning curve would therefore typically result in a full width that is half the size of the disparity scale. Thus, to get from the measured σ to neurophysiologically derived disparity scale requires a multiplier of 4.7.

To approximate this measure, we used ±2σ of the Gaussian fit; therefore, the disparity tuning widths described here are slightly smaller (0.85) than the best estimate described above. Moreover, the relationship between stimulus eccentricity and tuning width remains unclear, but eccentricity is likely to influence tuning width.

The disparity scale measures reported in V1 (Cumming and DeAngelis, 2001; Parker, 2007) range between ∼0.25 and 3°, while the range in the current study was from the imposed minimum of 0.10–2.2°, with an average of 0.34° (∼0.4° if the scale factor of 4.7 is used), indicating that many of the tuning curves are narrow relative to the neurophysiological range. A potential anatomical source of the difference could be the greater eye size and interocular separation of the eyes in humans as compared with macaque monkeys. If the neural apparatus of macaques and humans is identical in binocular performance, a widening of the interocular baseline in humans will improve their stereoacuity relative to macaques. By itself, this might explain a factor of 2. More detailed investigation of this difference may require investigation with state-of-the-art methods either recording directly from human neurons with intracranial electrodes (Decramer et al., 2019) or comparison of disparity pRF measured with fMRI and single-cell disparity tuning curves in the same nonhuman primate, equivalent to the comparison between receptive field and pRF by Klink et al. (2021).

Future studies could compare the fit of Gaussian and Gabor models to this type of 1D pRF model to determine experimentally whether this significantly affects the model tuning width, although the increased number of parameters may be challenging for current pRF methodology.

### Disparity tuning curve width is greater for nonzero disparity representations

We have demonstrated a second-order polynomial relationship between the magnitude of the preferred disparity and the width of the disparity tuning curve, shown in Figure 7. This relationship is bidirectional, i.e., it applies to both near- and far-tuned vertices and occurs for the correlated stimulus in all visual areas tested. One account of binocular sensitivity that explains this relationship is to consider disparity tuning in the context of the binocular energy model (Ohzawa et al., 1990), where disparity tuning is a property of complex binocular cells resulting from the linear combination of monocular inputs. To detect nonzero disparities, monocular receptive fields for the left and right eye display a position or phase displacement. The magnitude of the displacement is proportional to the preferred disparity, with large disparities necessitating a large displacement between the monocular receptive fields. As the distance (or phase shift) between the monocular receptive fields increases, the overall receptive field size must also increase to ensure overlap between the left and right monocular fields, which enables disparity sensitivity. It is this increase in the receptive field size which can account for a broader tuning curve width for cells with larger preferred disparities, as a larger spatial overlap between the monocular receptive fields allows a broader range of disparities to be encoded. When examining the relationship between the sharpness of disparity tuning and eccentricity across multiple studies and cortical areas, Anzai et al. (2011) showed a weak negative correlation. However, it is unclear if this effect holds “within” areas, as reported here. From a psychophysical point of view, the narrower disparity tuning for vertices with peak disparity close to the fixation plane is consistent with psychophysical studies that have found the best discrimination for stereo depth is for small changes in depth around the fixation plane (Schumer and Julesz, 1984).

### Stereoacuity thresholds correlate with the smallest disparity tuning widths in V1, but not extrastriate visual areas

The ability of cortical neurons to encode disparity information underlies the percept of stereopsis. As disparity sensitivity originates in V1 (Barlow et al., 1967; Nikara et al., 1968; Pettigrew et al., 1968) and V1 contains sharper disparity tuning curves when compared with extrastriate visual areas (DeAngelis and Newsome, 1999; Cumming and DeAngelis, 2001; Thomas et al., 2002; Tanabe et al., 2005; Parker, 2007), this area may be seen as the initial gating mechanism for subsequent disparity processing. To relate human cortical disparity selectivity to the perceptual limits of stereo perception, participants who took part in the imaging experiment also took part in a psychophysical task where perifoveal stereoacuity was assessed. We found the value of the 10th quantile for disparity tuning curving width, or disparity resolution limit, correlated with stereoacuity performance in area V1, but not in extrastriate visual areas. Neuronal responses in V1 do not reflect the percept of depth; V1 neurons do not encode relative disparity between neighboring visual features (Cumming and Parker, 1999), respond to false matches (Cumming and Parker, 2000), and are reliably driven by anticorrelated RDS stimuli which do not produce a depth percept (Cumming and Parker, 1997). However, the disparity selectivity of V1 can still influence the upstream processing of perceptually relevant disparity signals. For example, Nienborg et al. (2004) report a relationship in V1 between the receptive field size and disparity selectivity, with the size of the disparity receptive field limiting the acuity for disparity information. Area V1 may therefore act as a filter or gateway, where interindividual differences in the receptive field size and disparity tuning impose later limits on perceptual stereoacuity (Alvarez et al., 2021). This proposal for a gateway limitation at the level of V1 is also compatible with the neurophysiological observations of Prince et al. (2000), who found that V1 neurons often exhibit high sensitivity for small changes in stereoscopic depth, despite the lack of specialization of V1 neurons for disparity processing.

A limitation of the comparison between the pRF data and the psychophysical thresholds, however, is the difference in the size of disparity value used. The range of disparities used for the fMRI pRF mapping was kept relatively low at ±0.3° to ensure that participants were able to maintain fusion and to minimize any vergence eye movements. The pedestal used for the psychophysics was ±0.4°, with the discrimination around those values. Thus, there was little or no neural coverage of the largest values used in the psychophysical testing. Rather, the vertices with preferred disparities with magnitude >0.1° were used. It may be that, had larger disparities been used in the pRF mapping, a correlation between disparity tuning width and psychophysical performance may have been present outside of V1. In future studies it would be worth expanding the range of disparities presented in the scanner.

In summary, the findings presented here demonstrate the characteristics of disparity selectivity across the human visual cortex, with area V1 linked to the limits of stereoscopic performance and providing the gateway role of depth perception.
